# Prevalence of food insecurity and associated factors among households in Waliso town, Oromia, Ethiopia

**DOI:** 10.1186/s40795-023-00787-w

**Published:** 2023-11-27

**Authors:** Tariku Gurara Tulem, Mulatu Ayana Hordofa

**Affiliations:** 1 Waliso Town Health Office, Oromia Regional Health Bureau, Waliso, Ethiopia; 2 Department of Public Health, College of Medicine and Health Science, Ambo University, PO.BOX.240, Ambo, Ethiopia

**Keywords:** Associated factors food insecurity Waliso town

## Abstract

**Background:**

Food insecurity is the underlying cause of malnutrition. In addition, it is a condition where people lack consistent access to enough food for every person in a household to live an active and healthy life.

Recently, urban food insecurity has been on the top agenda in Ethiopia, as households prone to food insecurity are rising because of various factors. Hence, we aimed to assess the prevalence of food insecurity and associated factors among households in Waliso town, Oromia, Ethiopia.

**Methods:**

A community-based cross-sectional study design was used from December 17, 2021, to January 16, 2022, among 397 randomly selected households in Waliso town. Study participants were selected using a simple random sampling technique. Data collection was performed using a structured questionnaire, and data were entered using Epi Info 7 and exported to SPSS version 20 software for analysis. To identify factors associated with household food insecurity, multivariable binary logistic regression analyses were done. An adjusted odds ratio along with a 95% confidence interval were estimated to measure the strength of the association. In this study, the level of statistical significance was declared at p < 0.05.

**Results:**

The prevalence of household food insecurity was 63.4%, 95% CI (58.60%, 68.20%). Factors associated with household food insecurity were family sizes 6 + [AOR = 3.06, 95% CI: (1.35, 6.94)], dependents 1–2 [AOR = 2.62, 95% CI: (1.35, 5.10)], dependents 3 + [AOR = 3.07, 95% CI: (1.38, 7.40)], unemployed [AOR = 6.86, 95% CI: (2.76, 17.04)], self-employed [AOR = 3.27, 95% CI: (1.50, 7.14)], wage laborers [AOR = 13.01, 95% CI: (4.93, 34.38)], low wealth index [AOR = 4.08, 95% CI: (2.18, 7.64)], and a medium wealth index [AOR = 2.08, 95% CI: (1.18, 3.69)].

**Conclusion:**

Nearly two in three households were food insecure in Waliso Town. Large family sizes, a high dependency ratio, unemployment, low wage employment, and a low wealth index were significantly associated with food insecurity. Hence, the implementation of actions to improve household income and livelihoods, minimize dependency rates, by strengthen the use of family planning and encourage urban agriculture is vital to alleviate household food insecurity problems in the town.

**Supplementary Information:**

The online version contains supplementary material available at 10.1186/s40795-023-00787-w.

## Background

According to the Food and Agricultural Organization (FAO), food security occurs when all people have physical and economic access to adequate, safe, and nutritious food to meet their dietary needs and food preferences for an active and healthy life [1]. The four pillars of food security are availability, accessibility, utilization, and sustainability, and meeting them is considered a human right; however, a person is said to be in a state of food insecurity if these conditions are not fulfilled. Chronic food insecurity is the result of prolonged periods of poverty and a lack of assets. While transitory food insecurity is a transient phenomenon related to human-made and natural phenomena that result in instability in food prices, food production, or household incomes, in its worst form, it may yield famine [1, 2].

The issue of food insecurity is widespread, with 30% of the global population (2.37 billion) experiencing food insecurity in 2020. More than a third of them (799 million) were from Africa [3]. In Ethiopia, there were 64.7 million people who were food insecure from 2019–2021, which showed that more than half of the population was food insecure. The combination of the COVID-19 pandemic and ongoing war and instability in the country may be the reason [4]. Usually, people think that urban areas have fewer problems with food insecurity than rural areas, but this does not work because urban areas are most affected by profound differences between socioeconomic groups, ethnic groups, and religions [5].

Despite the lack of global-level data showing the magnitude of food insecurity disaggregated in urban and rural areas, there is evidence showing a high prevalence of food insecurity in urban areas. Thus, a study conducted in India revealed that nearly 2 in 5 households of urban slum dwellers were food insecure [6]. Likewise, a finding from South Africa showed that 61.4% of households were food insecure [7]. Moreover, in Ethiopia, the magnitude of household food insecurity varies across urban centers across the country. In 2015, a national-level longitudinal study revealed that nearly 1 in 5 small-town households and 1 in 7 large-town households were uncertain about having food for the family all year [8].

Another study in Hosanna town revealed that 41.7% of households, or 2 in 3, were food insecure [9]. The problem is multifaceted and caused by food price volatility [10], rapid urbanization, poverty [11], and the COVID-19 pandemic. It is further exacerbated by the rise in inflation, with the rate of inflation in Ethiopia reaching 35.1 percent in December 2021 and food prices rising by 41.6 percent, far above the norms observed in most African countries [12].

During periods of scarcity, “poor urban families often spend up to 70% of their earnings to purchase food, which makes them reduce spending on education and child care. Families consume fewer meals, sometimes not eating for whole days, and children stop going to school as parents save money on food [13]. Moreover, household food insecurity was associated with poor school attendance among adolescents. A systematic review showed that students from food-secure households were 57% less likely to be absent from school when compared with their counterparts [14]. Furthermore, a study in northwestern Ethiopia revealed that adolescent girls from food-insecure households are more vulnerable to anemia, which may cause cognitive impairment [15].

Food insecurity is a major contributor to chronic malnutrition, which contributes to child stunting and underweight [16]. In 2020, globally, 22%, or 149.2 million children under five, were stunted with more than half (53%) of them from Asia and more than one-third (41%) from Africa, 6.7%, or 45.4 million children, wasted, and 5.7% or 38.9 million overweight [17]. In Ethiopia, stunting among children under five was reduced from 58% in 2000 to 35.3% in 2020. However, nearly 5.8 million children under five years of age are stunted [18].

Malnutrition leads to poor health, impaired cognitive development, slow physical growth, reduced learning capacity, and lower adult work performance and efficiency [19]. Ethiopia loses 157.8–230.2 million United States dollars (USD) every year due to child wasting [20]. Likewise, malnutrition is a major cause of mortality among infants and children in Ethiopia, accounting for over 50% of all deaths [21].

The government of Ethiopia formulated a national food and nutrition policy in 2018 to guarantee the availability, accessibility, and use of a varied, safe, and nutritious food supply in the long term [22]. Despite this, the problem of urban food insecurity is rising in Ethiopia. However, the factors associated with urban food insecurity were not as thoroughly investigated as those associated with rural food insecurity in Ethiopia and Oromia. Therefore, this study aims to assess the prevalence of food insecurity and associated factors among households in Waliso town, Oromia, Ethiopia.

## Methods

A community-based cross-sectional study was employed from December 17, 2021, to January 16, 2022, at Waliso town in the Southwest Shewa zone, Oromia region, Central Ethiopia, which is 114 km southwest of Addis Ababa. Waliso town is located at 8°32° N and 37°58° E and is 2063 m above sea level. Its climate is Wiena Dega (temperate), and it rains three times a year. The main types of crops produced in the district surrounding the town are teff, wheat, maize, and inset. From the projection of the 2007 CSA report, Waliso town has 67,496 inhabitants, of which 33,643 were males and 33,853 were females in 2021, which is projected from the Ethiopian census conducted in 2007, and 14,673 households.

The town is classified into four administrative kebeles (lowest administrative units). The majority of Waliso town residents found food for their families by purchasing it from the market. While the majority of households are unemployed, wage laborers and self-employed individuals earn less to cope with the current high inflation in the country.

### Source population

All households in Waliso Town.

### Study population

All randomly selected households in Waliso town and household members aged > 18 years who were responsible for food purchase and preparation, preferably women from households in selected kebeles of Waliso town, were interviewed.

### Sample size determination

The sample size was determined by using the single population proportion formula.

$$\mathrm{n}=\frac{{\left(\mathrm{Z\alpha }/2\right)}^{2}\mathrm{p}\left(1-\mathrm{p}\right)}{{\mathrm{d}}^{2}}=\frac{{\left(1.96\right)}^{2}0.376\left(0.624\right)}{0.0025}=361$$

#### Assumptions

P = 37.6% (prevalence of household food insecurity among households in Woliata Sodo town) [23], Z/2 = critical value at 95% confidence level of certainty (1.96), d = margin of error (5%). Adding a 10% nonresponse rate, the final sample size (n) was 397.

The sample size for the second objective: The sample size for the second objective was calculated using EpiInfo7 software and by using three significantly associated variable percentages (P) and their respective adjusted odds ratios. Assuming power is 80% and Z/2 95% CI, then a 10% no response rate is added. Finally, the sample sizes found were sample size one (S1) = 346; sample size two (S2) = 382; and sample size three (S3) = 392, but all three sample sizes calculated for the second objective were less than the sample size calculated for the first objective, i.e., 397. Therefore, the sample size calculated for the first objective was also used for the second objective because it was the largest.

### Sampling procedure and technique

**Fig. 1:**
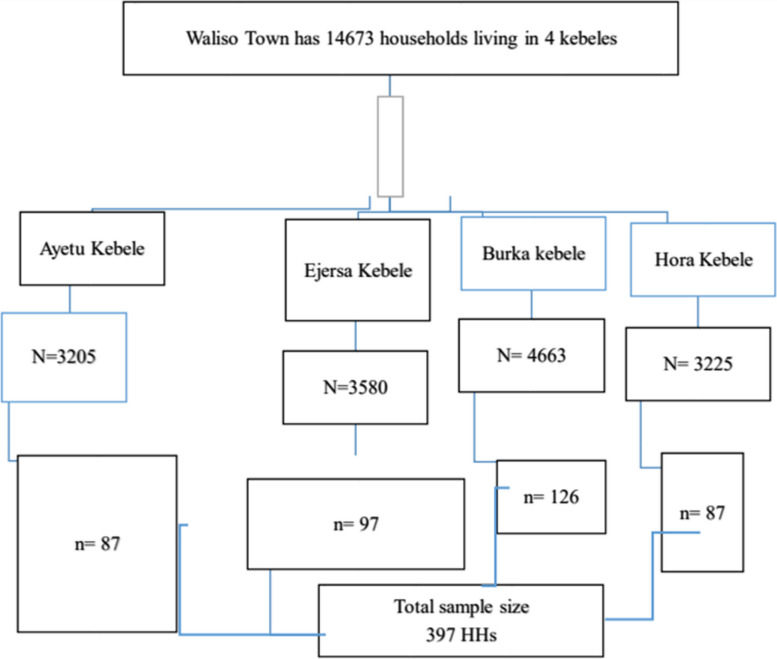
Sampling procedure used to assess the prevalence of household food insecurity and associated factors among residents of Waliso town, Oromia, Ethiopia, 2021/2022. Waliso town has four kebeles (the lowest administrative units), i.e. Ayetu, Burka, Ejersa, and Hora

All households that lived in Waliso town for more than six months were included in this study. There is a registration book containing the list of all households in each kebele, which is updated every six months and used for urban health extension program (family health) service provision based on their health needs, and the study participants for this study were selected by simple random sampling from this family health register in all kebeles in Waliso town. A computer program was used to select the needed sample population, and proportional allocation was employed for each kebele. After selecting the required sample by simple random sampling from the register, the data were collected house to house (Fig. 1).

### Data collection tools and techniques

A structured, interviewer-administered questionnaire translated into the regional language “Afaan Oromoo” and translated back to English by a language expert was adapted from the Household Food Insecurity Access Scale (HFIAS) version 3 from Food and Nutrition Technical Assistance (FANTA) and has nine occurrence questions that represent a generally increasing level of food insecurity (access) and has three domains. The first question is related to anxiety and uncertainty about household food supplies. The next three questions are about insufficient quality (variety and preferences of the types of food). The remaining five are questions about access to insufficient food intake and its physical consequences, and nine “frequency-of-occurrence” questions are asked as a follow-up to each occurrence question to determine how often the condition occurs [24].

Sociodemographic and wealth index questions were adapted from the Ethiopian Demographic and Health Survey. The household wealth index (HWI) questionnaire was constructed from household asset ownership questions that were adapted from the 2016 EDHS (Ethiopian Demographic and Health Survey) questionnaire [25] and categorized into three wealth categories: low wealth index (poor), medium wealth, and high wealth index (rich).

The data collection was performed by trained urban health extension professionals; two BSc health science professionals supervised the data collection activities, and the investigators monitored the overall data collection activities and procedures. The data collectors and supervisors were trained for two days on study variables, data collection, and interview techniques to minimize interviewer bias. The questionnaire was pretested on 5% of the sample (20 households) in the adjacent town of Waliso in Obi town. Then, the questionnaire was modified based on the pretest results; repetitive ideas and ambiguous questionnaires were corrected, and the modified questionnaire was used for the final data collection. Even if data collection overlapped with the festive period (Christmas), we interrupted the data collection process for five days during the Christmas celebration, and all dietary histories linked with the festive were excluded during data collection.

#### Data processing and analysis

After data collection, the questionnaire was checked for completeness and consistency. The data entry template was prepared and double-entered using Epi Info 7. The data were cleaned by limiting the values that would be entered, providing skipping patterns, and, after data entry, by using simple frequency, tabulating variable frequency, and sorting ascending and descending.

Then, the data were exported into SPSS version 20 for analysis. The household wealth index was determined by principal component analysis using asset ownership questions, easy-to-collect data on a household's ownership of selected assets, materials used for housing construction, and types of water access and sanitation facilities and categorized into poor, medium, and rich. First, univariable analysis was conducted using descriptive summaries such as frequencies, percentages, mean, standard deviations, and prevalence. Bivariable analyses were performed to identify candidate variables for a multivariable logistic regression model. Normality was checked for continuous variables using a histogram and Q–Q plot, and HH monthly income and HH monthly expenditure were not normally distributed, while HH age was normally distributed. Model fitness was checked using the Hosmer and Lemeshow test, and it was not significant (p > 0.05) except for the model being fit.

All explanatory variables that were associated with the outcome variable at a P value of 0.25 were considered candidates for multivariable logistic regression. Multicollinearity between different explanatory variables was checked using tolerance (tolerance 0.2) and the variable inflation factor (VIF > 5) [26]. Two independent variables, i.e., monthly household income (tolerance = 0.13, VIF = 7.53) and monthly household food expenditure (tolerance = 0.13, VIF = 7.56), were excluded from multivariable analysis with multicollinearity.

The association between the outcome variable and predictors was determined, and P < 0.05 was used to declare statistical significance. Adjusted odds ratios and 95% confidence intervals were used to measure the strength of the association.

### Study variables

#### Dependent variable

Household food security status: secure, insecure.

#### Independent variables

*Household food sources and access*: self-production, market purchase, donation, or transfer of money or food.

*Sociodemographic Factors and Economic Status*: Age, sex, marital status, occupation, educational status of the household head, family size and the number of dependents in the household, household monthly income, household monthly food expenditure, and household wealth index.

*Alcohol consumption and substance use*: alcohol consumption, chewing chat.

### Operational definitions

*HFIAS Score*: Replies on the nine HFIAS questions will have a minimum of zero and a maximum score of 27 (0–27), and HFIAS scores 0–1 were considered food secure, while those with HFIAS scores 2–27 were considered food insecure. Households’ food insecurity was also categorized according to their score, with HFIAS scores of 2–7, 8–14, and 15–27 indicating mild, moderate, and severe food insecurity, respectively [27]. Accordingly,

Food secure: HFIAS scores 0–1 were considered food secure.

Food insecure: HFIAS scores 2–27 were considered food insecure.

## Results

A total of 396 households responded to the study. The response rate was 99.7%. The mean (SD) age of the household heads was 39.7 (10.6) years. Among the 396 households that participated in the study, 298 (75.3%) were male-headed. The average family size of the households was 4.6.

A total of 251 (63.4%) of the household heads in the study were from the Oromo ethnic group, and more than half of the study participants, 199 (50.3%), were followers of the Orthodox Christian religion. The majority (290, or 73%) of household heads attended secondary school or lower. A total of 281 (71%) HH heads were married. Households’ wealth status as determined by the principal component analysis was 132 (33.3%), 144 (36.4%), and 120 (30.3%), low wealth status (poor), medium, and high wealth status (rich), respectively, using the asset ownership questions about their household wealth status. Moreover, 358 (90.4%) households in this study purchased their food from the market (Table 1).

**Table 1:** Socio-demographic, economic characteristics, and alcohol consumption of households, in Waliso Town, Ethiopia, 2021/2022 (*N* = 396)

Variables	Categories	Frequency	Percent (%)
Sex	Male	298	75.3
	Female	98	24.7
Age	18–29	55	13.9
	30–39	159	40.2
	40–49	117	29.5
	≥ 50	65	16.4
Family size	1–3	97	24.5
	4–5	208	52.5
	6 +	91	23.0
Dependent number	No dependent in Family	66	16.7
	1–2	244	61.6
	≥ 3	86	21.7
Ethnicity	Oromo	251	63.4
	Amhara	70	17.7
	Gurage	70	17.7
	Other	5	1.3
Religion	Orthodox	199	50.3
	Muslim	69	17.4
	Protestant	123	31.1
	Other	5	1.3
Marital status	Single	24	6.1
	Married	281	71.0
	Divorced	49	12.4
	Widowed	42	10.6
Educational status	No formal education	79	19.9
	Elementary (1–8)	104	26.3
	Secondary (9–12)	107	27.0
	Diploma and above	106	26.8
Employment	Un Employed	64	16.2
	Self Employed	91	23.0
	Wage laborer	73	18.4
	GO/NGO Employee	114	28.8
	Merchant	54	13.6
Monthly income	< 2929 ETB(57 USD)	175	44.2
	> 2929ETB(57 USD)	221	55.8
Food share of monthly Expenditure	< 50% of HH income	37	9.3
	50–75% of HH income	105	26.5
	> 75% of HH income	254	64.1
Had Bank Account	Yes	352	88.9
	No	44	11.1
Wealth Index	Poor	132	33.3
	Medium	144	36.4
	Rich	120	30.3
Food source	Self-Production	27	6.8
	Market Purchase	358	90.4
	Donation	11	2.8
Alcohol consumption	Yes	96	24.2
	No	300	75.8
chews chat	Yes	61	15.4
	No	335	84.6

*HH* Household, *USD* United States dollar, Ethnicity:—Other (Walene, Kebena, Silte), Religion:—Other (Wakefeta, Adventist)

### Prevalence of household food insecurity in Waliso Town

**Fig. 2:**
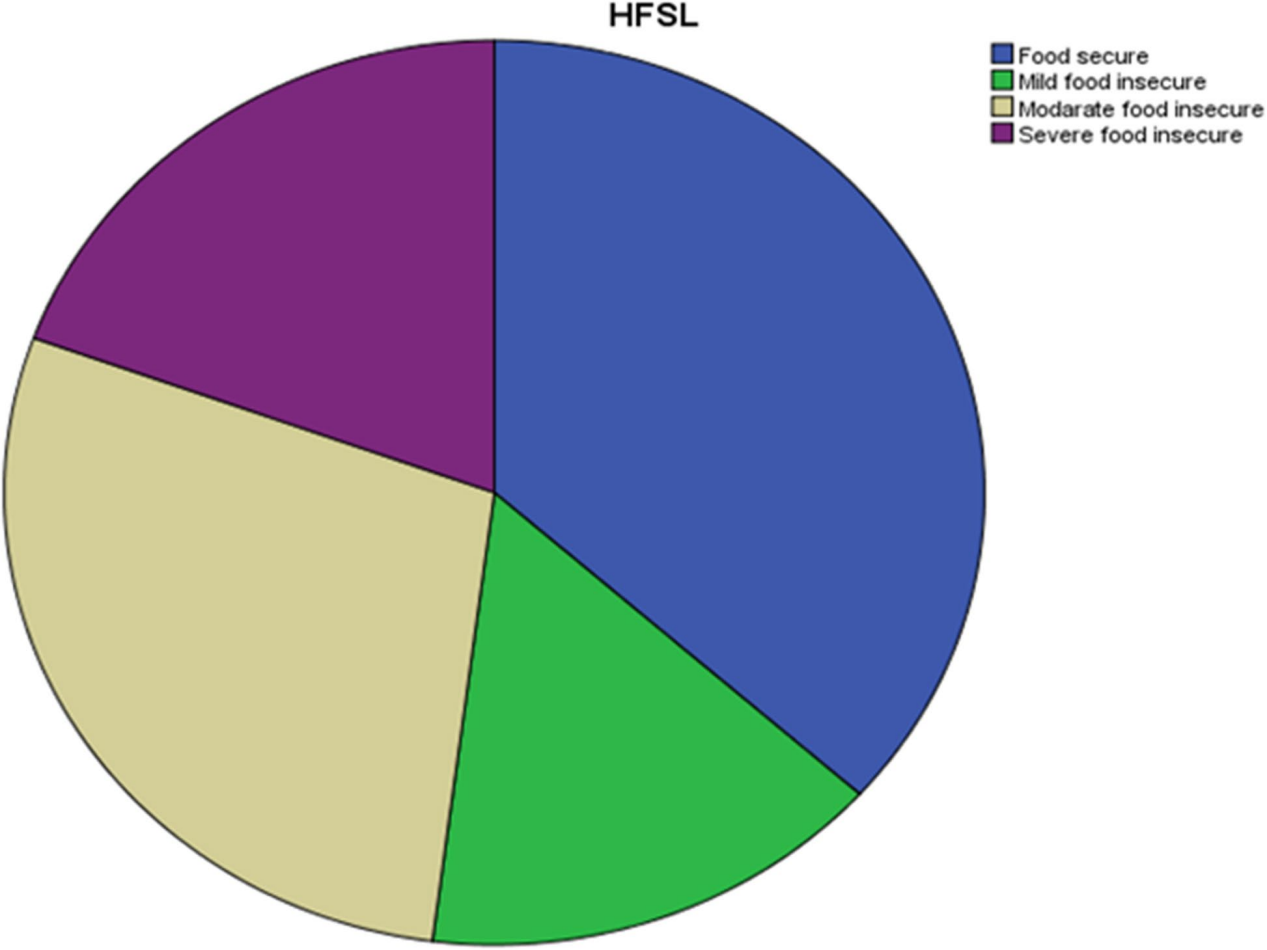
Prevalence of household food insecurity among households in Waliso Town, Oromia, Ethiopia, 2021/2022 (*n* = 396)

The prevalence of household food insecurity in the study area was 63.4% (95% CI: 58.6, 68.2). Household food insecurity was further categorized based on its severity. Accordingly, 15.4% 95% CI (11.9, 18.9), 28.5% 95% CI (23.7, 33.1), and 19.4% 95% CI (15.7, 23.7) were instituted to be mildly, moderately, and severely food insecure, respectively (Fig. 2).

Results of bivariable analyses on factors associated with household food insecurity in bivariable analyses, Household head age was not associated with household food insecurity in bivariable analysis. Household head alcohol consumption status and chat chewing status were also not satisfied (*p* < 0.25) (Table 2).

**Table 2:** Results of bivariate analyses on factors associated with household food insecurity among residents of Waliso town, Oromia, Ethiopia 2021/22 (N = 396)

Variables	Food Security Status		COR(95%CI)	*P*-value
	Insecure *N* (%)	Secured *N* (%)		
Sex				0.002*
Male	176(59.1)	122(40.9)	1.00	
Female	75(76.5)	23(23.5)	2.26(1.34, 3.81)	0.002
Age				0.222
18–29	38(69.1)	17(30.9)	1	
30–39	94(59.1)	65(40.9)	0.65(0.34, 1.24)	0.192
40–49	81(69.2)	36(30.8)	1.00(0.50, 2.01)	0.985
> = 50	38(58.5)	27(41.5)	0.63(0.30, 1.34)	0.230
Educational Level				0.001*
Not attend formal education	63(79.7)	16(20.3)	5.14 (2.63, 10.03)	0.001
Elementary	75(72.1)	29(27.9)	3.37(1.90, 6.00)	0.001
Secondary	67(62.6)	40(37.4)	2.19(1.26, 3.78)	0.005
Diploma and above	46(43.4)	60(56.6)	1.00	
Marital status				0.012*
Single	14(58.3)	10(41.7)	1.00	
Married	166(59.1)	115(40.9)	1.03(0.44, 2.4)	0.943
Divorced	40(81.6)	9(18.4)	3.18(1,07, 9.41)	0.037
Widowed	31(73.8)	11(26.2)	2.01(0.70, 5.83)	0.197
Family size				
(1–3)	52(53.6)	45(46.4)	1.00	0.072*
4–5	139(66.8)	69(33.2)	1.74(1.07, 2.85)	0.027
6 +	60(65.9)	31(34.1)	1.68(0.93, 3.02)	0.086
Dependent number				0.024*
No (0)	32(48.5)	34(51.5)	1.00	
(1–2)	163(66.8)	81(33.2)	2.14(1.23,3.71)	0.007
> 3	56(65.1)	30(34.9)	1.98(1.03, 3.82)	0.041
Employment status				.000*
Un Employed	51(79.7)	13(20.3)	5.29(2.34, 11.93)	.000*
Self Employed	61(67.0)	30(33.0)	2.74(1.37, 5.49)	.004*
Wage laborer	63(86.3)	10(13.7)	8.49(3.60, 20.03)	.000*
GO/NGO Employee	53(46.5)	61(53.5)	1.17(0.61, 2.25)	.636
Merchant	23(42.6)	31(57.4)	1.00	
Food sources				0.117*
Self-Production	14(51.9)	13(48.1)	1.00	
Market- Purchase	227(63.4)	131(36.6)	1.61(0.73, 3.53)	0.235
Donation	10(90.9)	1(9.1)	9.29(1.04, 82.96)	0.046
Had Bank Account				0.046*
Yes	217(61.6)	135(38.4)	1	
No	34(77.3)	10(22.7)	2.12(1.012, 4.420)	
Consumes Alcohol				0.799
Yes	62(64.6)	34(35.4)	1	
No	198(63)	111(37)	1(0.66, 1.73)	
Chews chat				0.631
Yes	37(60.7)	24(39.3)	1	
No	214(63.9)	121(36.1)	1.15(0.655, 2.01)	
Wealth index				0.001*
Poor	104(78.8%)	28(21.2%)	5.03(2.89, 8.73)	0.001*
Medium	96(66.7%)	48(33.3%)	2.71(1.64, 4.74)	0.001*
Rich	51(42.5%)	69(57.5%)	1.00	

^*^P < 0.25; *COR* crude odds ratio, *CI* confidence interval

### Factors associated with household food insecurity in multivariable analysis

Multivariable logistic regression analysis revealed that household head employment status was significantly associated with household food insecurity in Waliso Town. Households headed by the unemployed were seven times (AOR = 6.86, 95% CI: 2.76, 17.04) more likely to be food insecure than HHs headed by merchants; HHs headed by the self-employed were three times (AOR = 3.27, 95% CI: 1.50, 7.14) more likely to be food insecure than their counterparts. Additionally, households headed by wage laborers were thirteen times (AOR = 13.01, 95% CI: 4.93, 34.38) more likely to be food insecure than households headed by merchants.

Moreover, household family size had a statistically significant association with household food insecurity in the study area; that is, households with greater than six family sizes were 3 times (AOR = 3.06, 95% CI: 1.35, 6.94) more likely to be food insecure than their counterparts. Likewise, the presence of a dependent family member in the household was significantly associated with household food insecurity in Waliso Town. HHs with 1–2 dependents were (AOR = 2.62, 95% CI: 1.35, 5.10) more likely to be food insecure than HHs who had no dependents (children < 15 years old and/or elders > 65 years old) in a household. Households that had more than three (> 3) dependents were three times (AOR = 3.07, 95% CI: 1.28, 7.40) more likely to be food insecure than households that did not have dependent family members.

Furthermore, the household wealth index revealed a statistically significant association with household food insecurity in Waliso Town. Households with a low wealth index (poor) were 4 times (AOR = 4.08 95% CI: 2.18, 7.64) more likely to be food insecure than households with a high wealth index (rich), and households with a medium wealth index were 2 times (AOR = 2.08, 95% CI: 1.18, 3.69) more likely to be food insecure than their counterparts (Table 3).

**Table 3:** Multivariable logistic regression analysis of factors associated with household food insecurity Waliso Town, Oromia, Ethiopia, 2021/2022 (*N* = 396)

Variables		Food Security Status		**COR(95%CI)**	**AOR(95%CI)**	***P*-Value**
		Insecure N (%)	Secured N (%)			
Family size	(1–3)	52(53.6)	45(46.4)	1.00	1.00	
	4–5	139(66.8)	69(33.2)	1.74(1.07, 2.85)	1.82 (1.00, 3.32)	0.051
	6 +	60(65.9)	31(34.1)	1.68(0.93, 3.02)	3.06 (1.35, 6.94)	0.007*
Dependent nu ember	No (0)	32(48.5)	34(51.5)	1.00	1.00	
	(1–2)	163(66.8)	81(33.2)	2.14(1.23,3.71)	2.62 (1.35, 5.10)	0.004*
	> 3	56(65.1)	30(34.9)	1.98(1.03, 3.82)	3.07 (1.28, 7.40)	0.012*
Employment	Un Employed	51(79.7)	13(20.3)	5.29(2.34, 11.93)	6.86 (2.76, 17.04)	0.001**
	Self Employed	61(67.0)	30(33.0)	2.74(1.37, 5.49)	3.27 (1.50, 7.14)	0.003*
	Wage laborer	63(86.3)	10(13.7)	8.49(3.60, 20.03)	13.01 (4.93, 34.38)	0.001**
	GO/NGO Employee	53(46.5)	61(53.5)	1.17(0.61, 2.25)	1.35 (0.68, 2.70)	0.393
	Merchant	23(42.6)	31(57.4)	1.00	1.00	
Wealth Index	Poor	104(78.8%)	28(21.2%)	5.03(2.89, 8.73)	4.08 (2.18, 7.64)	0.001**
	Medium	96(66.7%)	48(33.3%)	2.71(1.64, 4.74)	2.08 (1.18, 3.69)	0.012*
	Rich	51(42.5%)	69(57.5%)	1.00	1.00	

*AOR* Adjusted odds ratio, *CI* confidence interval

Significant at * *P* < 0.05, ** *p* < 0.001

^*^Shows significant association for tabulated variables

## Discussion

This study assessed the prevalence of household food insecurity and associated factors among residents of Waliso Town. The prevalence of household food insecurity in Waliso Town was 63.4% (95% CI: 58.6, 68.2). This study indicated that approximately two-thirds of households in Waliso town were food insecure. The findings of this study were consistent with a study conducted in East Africa [28]. It was also aligned with the study conducted in Addis Ababa [29]. Similarly, it was aligned with the study conducted in Gambella town [30].

However, it was higher than the findings of studies conducted in India [6], Nigeria’s Ibadan metropolis [31], Ethiopia, Hosanna town [9], and Woliata Sodo town [23]. This may be because more than 90% of households in the study area found food for their families by purchasing it from the market. There was high food inflation in Ethiopia. During periods of high food prices, the purchasing power of poor households diminishes. This high prevalence might be attributed to the current high food price inflation, the implications of security problems in different parts of the country, and the war in northern parts, which hindered farming activity and led to low food grain production and productivity. Food grains have not been easily moved from place to place and marketed to buyers.

However, the prevalence of household food insecurity in this study was less than the prevalence of household food insecurity identified in North India [32]. This lower prevalence may be due to all study participants being urban slum populations in North India, but all randomly selected households were included in the study in Waliso town, irrespective of their slum status. Moreover, the prevalence of household food insecurity in Waliso town was also lower when compared with the study conducted in other African cities: South Africa, Cape Town, Kampala, Accra, Ghana, and Nairobi. This higher prevalence in those cities may be because Nairobi, Kampala, and Accra are the capitals, and the prices of food and living are high when compared with Waliso, which is a small town [33–36]. It was also lower when compared to the study conducted in Jimma, Ethiopia. This might be because the study population in Jima was pensioners, which was different from this study, which used all randomly selected sampled households without any preconditions of economic status or occupation.

Household head Employment was significantly associated with HH food insecurity in this study, and households headed by unemployed household heads were more food insecure than households headed by merchant household heads. This might be because the majority of households in this study (> 90%) find food by purchasing it from the market, in addition to the fact that household head employment is the main source of earnings. Similarly, households headed by daily wage employees were more food insecure than their counterparts. This might be because daily wage laborers earn low and unreliable incomes, which lowers their purchasing power. Households may face food insecurity when the household head is engaged in low-income employment. The findings of this study were consistent with other studies in urban Ethiopia, including Addis Ababa and Woliata Sodo [23, 37].

Additionally, having a large family size showed a statistically significant association with household food insecurity. Households with family sizes greater than six family members were more likely to be food insecure than households with fewer than three family members. This shows that having a large family size is a risk factor for household food insecurity, and this might be because sharing meals between extended families might affect the quantity and quality of meals consumed by children and women. This finding was consistent with other studies conducted in Uganda, Kampala [33], the South African city of Tshwane [38], and Ethiopia [29, 39].

Likewise, having dependent family members was significantly associated with household food insecurity, and households that had dependent family members were more food insecure than their counterparts. This shows that having dependent family members is a risk factor for household food insecurity; this might be because dependents do not contribute to income-generating food purchases while they share HH’s diet and costs for dependents, such as school fees, clothing, and milk for children, and health care costs for elderly dependent family members in addition. This finding was consistent with the studies conducted in India [32], Woliata Sodo Town, and Jima Town [23, 40].

Furthermore, households with a low wealth index (poor) were more likely to be food insecure than their counterparts. This might be explained by the fact that poor urban households might be unable to purchase adequate food to satisfy their household feeding needs due to the current high food inflation in Ethiopia, poor households’ inadequate food accessibility, and low purchasing power. The findings of this study were consistent with those studies conducted in Sub-Saharan Africa [39, 41], East Africa [28], Areka Town, southern Ethiopia, Addis Ababa, and central Ethiopia [37, 42]. This shows that the poor people living in Waliso town have difficulty ensuring their food security, similar to those living in other African cities and other Ethiopian cities.

However, household head gender did not show a statistically significant association with household food insecurity in this study. This might be because more than 75% of household heads in this study were male. However, the gender of the HH head was significantly associated with household food insecurity in studies conducted in Brazil [43] and Ethiopia [44].

Similarly, household head educational attainment was not associated with household food insecurity in Waliso Town. This might be due to the low payment an educated person earns, or it might be due to the current high inflation in the country. However, household head educational attainment showed a statistically significant association with food insecurity in studies conducted in Nigeria [31], Kenya [41], and Ethiopia [37, 45].

### Limitations of this study

The overlap of the study period with the harvest season might underestimate the occurrence of household food insecurity in the study area. As the study design of this study was a cross-sectional study, it is not possible to determine cause effects or relationships over time.

## Conclusion

Currently, urban food insecurity is on the top agenda in Ethiopia. Nearly two in three households were food insecure in Waliso town. The household food insecurity problem in Waliso town is due to a combination of factors. Large family sizes, unemployment, low wage employment, and a low wealth index (poor) and medium were significantly associated with household food insecurity in the town. Hence, strengthening actions to reduce the number of dependents in the family by strengthening the use of family planning to improve household income and livelihoods, utilize urban agriculture, and encourage the community to grow vegetables and fruits and raise livestock in their compound so that they become food self-sufficient is crucial.

### Supplementary Information

**Additional file 1:** **Appendix I.** Informed consent form. **Appendix II.** Declaration of the voluntary consent form. **Appendix III.** English version questionnaires.

## Data Availability

All the data relevant to the manuscript are reported in tables. The raw data can be accessed from the corresponding author upon request.

## Abbreviations

AOR: Adjusted odds ratio
CI: Confidence interval
COR: Crude Odds Ratio
FAN TA: Food and Nutrition Technical Assistance
FI: Food insecurity
HFIAS: Household food insecurity assessment scale
HFI: Household food insecurity
NGO: Non-Governmental Organization
UFI: Urban Food Insecurity, United Nations
USD: United States of America dollar
