# Supplementary material for: Prevalence of food insecurity and associated factors among households in Waliso town, Oromia, Ethiopia
Source: BMC Nutr. 2023 Nov 27;9:139. doi: 10.1186/s40795-023-00787-w (PMC10683113; doi:10.1186/s40795-023-00787-w)
Supplement: Supplementary file 1 — Additional file 1: Appendix I. Informed consent form. Appendix II. Declaration of the voluntary consent form. Appendix III. English version questionnaires. [file 40795_2023_787_MOESM1_ESM.pdf]

Appendices

Appendix I: - Informed consent form

Investigator: - Tariku Gurara Tulem (1Waliso Town Health Office, Oromia Regional Health Bureau, Waliso, Ethiopia)

Phone +251 926678262, Email tarikutulem@gmail.com

Advisor: - Mulatu Ayana Hordofa (Department of Public Health, College of Medicine and Health Science, Ambo University, PO.BOX.240: Ambo, Ethiopia

Phone: +251911549352/ +251920769735

Email: mulatuayana@gmail.com

Research team:-

Before starting any question please remember the following

Introduce yourself, give clarification at all times and seek their willingness for this study

Good Morning/Good Afternoons

My name is ____________and I am working with post-graduate student of Ambo University. Today I come to visit your home to ask you for information about the Magnitude of food insecurity and associated factors in your household. The interview will take about 30 to 45 minutes the interviewer will take notes of the response.

Risk of Participation:-

There is no anticipated risk involved with this interview. If there is an unclear question or issue you can ask for elaboration to properly understand and respond accordingly. You do not have to answer any question(s) if you do not want.

Benefits and Compensation of participation:-

Your participation in this study and the answers you give will be beneficial for the community as a whole, the information collected will help the government to identify the relevant variables of interest for nutrition sensitive intervention and to improve the health status of the community. You will not receive monetary compensation for this interview.

Privacy:-All of the answers you give will be confidential and will not be shared with anyone other than a member of our research team. Your name will not be recorded on this questionnaire and the information sheet will be kept locked in the file cabinet.

Voluntary Participation:-This interview is voluntary, you can discuss as much as you like or as little as you like. You do not have to answer any questions that you do not feel comfortable with. You can stop the interview at any time without giving any reason. The decision not to participate or to withdraw will not affect any aspects of your community life and your relationship with the university or any stakeholders associated with this study. If it is unclear, or you need further information the investigator will be happy to provide it.

If you are willing to be part of the study, we will be continuing. Otherwise, we can stop.

Appendix II: - Declaration of the voluntary consent form

Respondent agreement: are you willing to be part of the study? 1. Yes (proceed) 2. No (stop)

I have understood the explanation given to me. I have agreed that I shall enroll in the study.

(Make a sign: ----------------------------- Date -------------------)

Appendix III: - English version questionnaires

The questionnaire prepared for households in Waliso Town

Title: - Prevalence of household food Insecurity and Associated Factors among Residents of Waliso town, Central Ethiopia 2021

Questionnaire serial number (code): _______ Date of data collection: __________________

Town ________ Kebele ______house number: ____________

Data collector's **Name: ______________________ Signature: ____**

Supervisor's **Name: _________________________ Signature: ____**

I. Questions about general households' Economic and demographic characteristics

SN	Questions	Respond options	Code
101	Who is the head of the household?	1. Father<br>2. Mother<br>3. Other specify _______	|__|
102	What is the age of the household head?<br>(complete in years)	___________Years	|__|
103	What is the head of the household religion?	1. Orthodox<br>2. Protestant<br>3. Muslim<br>4. Catholic<br>5. Others __	|___|

104	What is the head of the household`s ethnicity?	1. Oromo<br>2. Amhara<br>3. Gurage<br>4. Other	|___|
105	What is the highest grade the household head had completed?	1. Can't read and write<br>2. Can read and write<br>3. Elementary school<br>4. Secondary school<br>5. Diploma and above	|___|
106	What is the employment status of the household head?	1. Unemployed<br>2. Self-employed<br>3. Wage labor<br>4. Pensioner<br>5. Government employee(GO)<br>6. NGO Employee<br>7. Merchant<br>8. Other (specify):_____	|__|
107	What is the marital status of the household head?	1. Single<br>2. Married<br>3. Divorced<br>4. Widowed	|__|
108	How many family members do you have?	_______________	|___|
109	How many children ≤15years and elders ≥65years in the household?	____________	
110	Who is the owner of the house you live in?	1. It is our private house<br>2. Kebele/gov`t rent<br>3. Rent from a private owner<br>4. Other Specify ______	|___|

111	Average monthly household income (*put the sum of incomes contributed by family members*)	____________Birr	|__|
112	What is the average monthly food expenditure of your Household?	____________Birr	|__|
113	In the last six months what was the main source of your household food consumption?	1. Purchased from market<br>2. From own production<br>3. Donation/from any agent<br>4. Others (specify)________	|___|
114	Does any member of this household have a bank account?	1. Yes<br>2. No	|__|
115	Does your husband drinks alcohol currently?	1. Yes 2. No	|__|
116	If yes how often does he drinks?<br>How often do you have a drink containing alcohol?	1. Monthly or less<br>2. 2 - 4 times per month<br>3. 2 - 3 times per week<br>4. 4+ times per week	|__|
117	Does your husband chew chat Currently?	1. Yes 2. No	|__|
118	If yes how often does he chew chat?	1. Monthly or less<br>2. 2 - 4 times per month<br>3. 2 - 3 times per week<br>4. 4+ times per week	|__|
119	Material their house made up of, observe?	1. Concrete or wood<br>2. mud	|__|
120	Roof material observe?	1. tiles or galvanized iron or concrete<br>2. Mud	|__|
121	What is your drinking water source?	1. Pipe in the dwelling<br>2. Pipe in the yard<br>3. Pipe to a neighbor	

		4. Public tap<br>5. Other ______		
122	What kind of toilet facility do your family members use?		Yes	No
		1. private pour-flush latrine		
		2. private pit latrine		
		3. communal latrine		
		4. Open field		
123	Does your household own?		Yes	No
		1. Electricity		
		2. Television		
		3. Radio		
		4. A none-mobile telephone		
		5. Computer		
		6. Refrigerators.		
		7. Modern Beds,		
		8. tables		
		9. Chair		
124	Does any member of your family member own:-		Yes	No
		1. A watch		
		2. A mobile phone		
		3. Bicycle		
		4. A motorcycle		
		5. An animal-drawn cart		
		6. Car/Truck		

II. Occurrence and Frequency of Household Food Insecurity questioner

S.N	Question	Response options	Code
201	In the past four weeks, did you ***worry*** that your HH would not have enough food?	0 = No (skip to Q2)<br>1=Yes	|___|
201a	How often did this happen?	1 = Rarely (once or twice in the last 4 weeks)<br>2 = Sometimes (3-10 times)<br>3 = Often (more than ten times)	|___|
202	In the past four weeks, were you or any HH member ***not able to eat the kinds of foods you preferred*** because of a lack of resources?	0 = No (skip to Q3)<br>1=Yes	|___|
202a	How often did this happen?	1 = Rarely (once or twice)<br>2 = Sometimes (3-10 times)<br>3 = Often (more than ten times)	|___|
203	In the past four weeks, did you or any HH member have to eat ***a limited variety of foods*** due to a lack of resources?	0 No (skip to Q 4)<br>1 = Yes	|__|
203a	How often did this happen?	1 = Rarely (once or twice)<br>2 = Sometimes (3-10 times)<br>3 = Often (more than ten times	|___|
204	In the past four weeks, did you or any HH member have to ***eat some foods that you did not want to eat*** because of a lack of resources to obtain other types of food?	0 = No (skip to Q5)<br>1 = Yes	|___|
204a	How often did this happen?	1 = Rarely (once or twice)<br>2 = Sometimes (3-10 times)<br>3 = Often (more than ten times)	|___|
205	In the past four weeks, did you or any HH ember have to ***eat a smaller meal than you felt*** you needed because there was not enough food?	0 = No (skip to Q6)<br>1 = Yes	|___|

205a	How often did this happen?	1 = Rarely (once or twice)<br>2 = Sometimes (3-10 times)<br>3 = Often (more than ten times)	|___|
206	In the past four weeks, did you or any other HH member have to eat ***fewer meals in a day*** because there was not enough food?	0 = No (skip to Q7)<br>1 =Yes	|___|
206a	How often did this happen?	1 = Rarely (once or twice)<br>2 = Sometimes (3-10 times)<br>3 = Often (more than ten times)	|___|
207	In the past four weeks, was there ***ever no food to eat of any kind*** in your HH because of a lack of resources to get food	0 = No (skip to Q8)<br>1 = Yes	|___|
207a	How often did this happen?	1 = Rarely (once or twice)<br>2 = Sometimes (3-10 times)<br>3 = Often (more than ten times)	|___|
208	In the past four weeks, did you or any HH member ***go to sleep at night hungry*** because there was not enough food?	0 = No skip to Q 9)<br>1 = Yes	|___|
208a	How often did this happen?	1 = Rarely (once or twice)<br>2 = Sometimes (3-10 times)<br>3 = Often (more than ten times)	|___|
209	In the past four weeks, did you or any HH member ***go a whole day and night without eating anything*** because there was not enough food?	0 = No (skip to section 3)<br>1 = Yes	|___|
209a	How often did this happen?	1 = Rarely (once or twice)<br>2 = Sometimes (3-10 times)<br>3 = Often (more than ten times)	|___|

Thank You!
